# Response Predictors in Chronic Migraine: Medication Overuse and Depressive Symptoms Negatively Impact Onabotulinumtoxin-A Treatment

**DOI:** 10.3389/fneur.2019.00678

**Published:** 2019-07-10

**Authors:** Francesca Schiano di Cola, Salvatore Caratozzolo, Paolo Liberini, Renata Rao, Alessandro Padovani

**Affiliations:** ^1^Neurology Unit, Department of Clinical and Experimental Sciences, University of Brescia, Brescia, Italy; ^2^Neurology Unit, Spedali Civili Hospital, Brescia, Italy

**Keywords:** chronic migraine, response predictors, efficacy, onabotulinumtoxin-A, depression, medication overuse

## Abstract

**Background:** Despite numerous studies that have investigated clinical, radiological, and biochemical response predictors, the clinical profile of those patients who might benefit from OnabotulinumtoxinA is still missing. The aim of the present study was to identify potential OnabotulinumtoxinA response predictors among several clinical characteristics and confirm OnabotulinumtoxinA efficacy and safety in chronic migraine (CM) prevention.

**Methods:** The study was conducted at the Headache Center—Neurology Clinic—Spedali Civili Hospital of Brescia. Eighty-four consecutive CM patients were enrolled, with a mean age of 48 years (SD 9.7) and a mean disease duration of 10.1 years (SD 6.6). The mean reported headache-days frequency was 22.5 (SD 5.9) per month, while the mean number of severe headache-days was 15.2 (SD 8.9) with a mean monthly medication intake of 33.2 (SD 5.6). The clinical characteristics analyzed as potential response predictors were: gender, disease duration, migraine characteristics (location, side constancy, unilateral autonomic and neurovegetative symptoms), previous prophylactic treatments, add-on therapies, withdrawal therapies, psychiatric (anxiety and depression symptoms) comorbidities and medication overuse.

**Results:** A significant reduction from baseline to 3, 6, 9, and 12 month treatment cycles in total headache days, high intensity headache days and triptans consumption per month was found. Depressive symptoms and medication overuse negatively predicted OnabotulinumtoxinA outcome.

**Conclusions:** Our results confirm the efficacy and safety of OnabotulinumtoxinA in CM. Depressive comorbidity and medication overuse, among all clinical variables, were the only significant response predictors. Such findings provide interesting insights regarding patients selection for OnabotulinumtoxinA treatment as, with the introduction of anti calcitonin gene-related (CGRP) monoclonal antibodies, clinicians will have to thoroughly judge and tailor among the many available therapeutic options now available. Future research might be needed to confirm our findings, in particular for its therapeutic implications.

## Background

Chronic migraine (CM), a headache occurring on ≥15 days/month (with migraine characteristics on ≥8 days/month) for at least 3 months (1), affects ~1.4–2.2% of adults and untold millions worldwide (2), causing greater disability compared to episodic migraine (EM) and significantly impacting quality of life (3). CM sufferers, compared to EM, tend to report lower levels of household income and full-time employment and are more likely to be occupationally disabled (4). Moreover, EM and CM are associated with a significant number of systemic and psychiatric comorbidities such as obesity, irritable bowel syndrome and autoimmune, respiratory, vascular, sleep and affective disorders (4–9). In particular, major depression, anxiety and post-traumatic stress disorder were found to be more frequent in CM than in patients with EM, as well as being significant risk factors for migraine chronification (9).

Such an individual, health related and economic burden imposes the need for a mandatory safe and effective treatment. OnabotulinumtoxinA was the first, and in many countries still the only, treatment specifically and selectively approved for the prophylaxis of CM in adults. Its approval, safety and efficacy were based on the results of the Phase III Research Evaluating Migraine Prophylaxis Therapy (PREEMPT) studies (10, 11)—two large, randomized double-blind, placebo-controlled trials—and the recent Chronic migraine OnabotulinuMtoxinA Prolonged Efficacy open Label (COMPEL) study (12)—a multicenter, open-label long-term prospective study. These studies demonstrated that OnabotulinumtoxinA treatment not only significantly reduced the frequency of headache days but also showed highly significant improvements in multiple headache symptom measures and in patients' self-perceived quality of life. Besides, patients not responding to the first treatment cycle may well-respond to up to two subsequent cycles (13). These results were confirmed by several real-life studies (14–16). Moreover, since its approval in Italy in 2013, prophylaxis with OnabotulinumtoxinA is perceived by Headache specialists as generally effective and safe, with a high level of compliance to recent recommendations (17). OnabotulinumtoxinA efficacy has been proven even in the context of CM associated with medication overuse, a frequent complication found in chronic migraneurs, encumbered by enormous treatments failure rates (18, 19).

Several studies have been conducted, over the past few years, searching for imaging, molecular and clinical response predictors. A recent MRI study conducted by Hubbar et al. (20) revealed that OnabotulinumtoxinA responders showed significant cortical thickening in the right primary somatosensory cortex, anterior insula, left superior temporal gyrus, and pars opercularis compared to non-responders, whereas Bumb et al. (21) did not find any significant difference in terms of white matter lesions between responders and non-responders. Increased interictal plasma levels of calcitonin gene-related peptide (CGRP) (22, 23), vasoactive intestinal peptide (VIP) (22) and pentraxin 3 (PTX3) (23)—all markers of trigeminal and parasympathetic activation—have been associated with better responses to OnabotulintoxinA, whereas decreased interictal CGRP salivary levels (24) were found in response to OnabotulinumtoxinA. Clinical variables have been the more thoroughly investigated, being more easily obtainable, as potential response predictors. Imploding (25), strictly unilateral pain (26, 27), unilateral autonomic symptoms (eyelid edema, tearing, nasal congestion, etc.) (27), short disease duration (26, 28), pericranial muscle tenderness (29, 30), ocular-type headache (31), and younger age (32) have all been associated with better clinical responses, though such findings have not been constantly replicated. Despite the number of studies that have focused on OnabotulinumtoxinA response predictors, the clinical phenotype of patients who might benefit from OnabotulinumtoxinA is still missing.

The aim of the present study was to identify potential OnabotulinumtoxinA response predictors among several clinical characteristics and confirm OnabotulinumtoxinA efficacy and safety in CM prevention.

## Materials and Methods

### Subjects

The study was conducted at the Headache Center—Neurology Clinic at the Spedali Civili Hospital of Brescia. We prospectively evaluated all adults aged 18–65 years with CM, with or without a diagnosis of medical overuse, between January 2015 and October 2018. Diagnosis was made according to the ICHD III beta criteria (33). All patients enrolled had failed to respond to at least two different classes of prophylactic treatments. Exclusion criteria were the following: hypersensitivity to OnabotulinumtoxinA or to any of the excipients, presence of infection at the proposed injection sites, presence of neuromuscular (e.g., myasthenia gravis or Lambert-Eaton Syndrome) and/or peripheral motor neuropathic diseases (e.g., amyotrophic lateral sclerosis or motor neuropathy), and pregnancy. We collected data concerning demographics, migraine characteristics (location, side constancy, presence of unilateral autonomic, and neuro vegetative symptoms), disease duration, previous prophylaxis, and withdrawal therapies (within the preceding 6 months), add-on therapies, headache days for month (distinguished in days with mild pain and moderate-intense pain), symptomatic drugs overuse (triptans, NSAIDs and combination analgesics), and systemic and psychiatric comorbidities (depression and anxiety). Patients were considered positive for depressive symptoms if they were experiencing, at baseline, a depressed mood together with at least three of the following symptoms: diminished interest or pleasure in their daily activities, significant weight loss, fatigue, feelings of worthlessness, and diminished ability to concentrate (33). Patients were considered positive for anxiety symptoms if they were experiencing, at baseline, excessive anxiety and worry, together with at least three of the following symptoms: restlessness, fatigue, impaired concentration, irritability, increased muscle aches or soreness, difficulty sleeping (33). At each subsequent evaluation, every 3 months, data on the frequency and intensity of headaches, use of symptomatic drugs, and the occurrence of side effects (by analysis of headache diary) was gathered. Migraine induced disability was assessed using the Migraine Disability Assessment Score Questionnaire (MIDAS) and the Short Form-36 (SF-36) at baseline, 3, 6, 9, and 12 months after treatment onset.

### Methods

Patients were injected with OnabotulinumtoxinA according to the PREEMPT protocol, after their informed consent for treatment with OnabotulinumtoxinA. We administrated 155 units intramuscularly using a 29 gauge needle as 0.1 ml (5 U) in 31 sites around the head and neck, divided across seven specific areas: corrugator 10 U, procerus 5 U, frontalis 20 U, temporalis 40 U, occipitalis 30 U, cervical paraspinal muscle group 20 U, and trapezius 30 U. At the investigator's discretion, additional units, up to 40, could be administered into the temporalis, occipitalis and/or trapezius muscle using a follow-the-pain strategy. The decision on any additional doses and locations were based on the patient's report of a usual location or predominant pain and the clinician's best judgement of the potential benefit of additional doses in the specified muscles. The maximum total dose we actually administered was 175 U (additional 20 U) in 35 sites, accordingly to a patient's pain and/or side localization.

### Statistical Analysis

Statistical analyses were performed with IBM SPSS Statistics 25.0 software for Windows (SPSS Inc., Chicago, IL, USA).

Regarding response predictors, we only analyzed data from patients who had completed at least three treatment cycles. For continuous variables (disease duration, number of previous prophylactic treatments) a linear regression analysis was conducted, whereas for ordinal variables (gender, location, side constancy, presence of unilateral autonomic and neurovegetative symptom, withdrawal therapy, add-on therapies, medication overuse, depressive, and anxiety symptoms) chi-square analyses were conducted.

A one-way repeated measures ANOVA was conducted to test whether there were statistically significant differences in the days of headache per month (overall and subdivided in days of high and low intensity), analgesics' consumption per month (overall and subdivided according to the different type of analgesic, i.e., NSAIDs, triptans or combination analgesics), and MIDAS and SF-36 scores from baseline to 3, 6, 9, and 12 months of treatment. A significant difference was set to be at *p* < 0.05.

## Results

**Figure 1:**
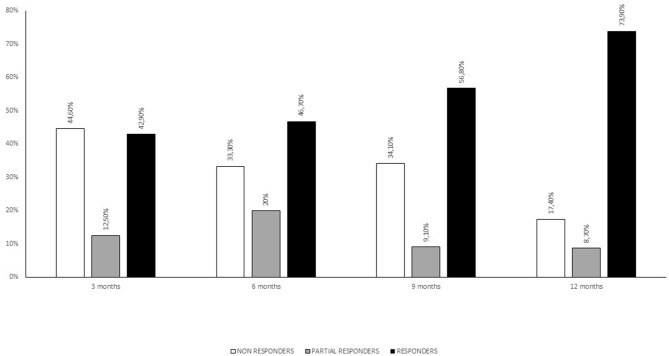
Responders rates at 3, 6, 9, and 12 months of treatment.

Eighty-four consecutive patients were enrolled (73% females) with a mean age of 48 years (SD 9.7) and a mean disease duration (duration since CM diagnosis) of 10.1 years (SD 6.6). The mean patient-estimated headache-days frequency was 22.5 days (SD 5.9) per month, while the mean number of days with severe headache (NRS ≥ 6) was 15.2 (SD 8.9). Fifty-five patients (65.5%) displayed medication overuse. Twenty-five patients (28%) had a history of withdrawal therapy in the 6 months preceding OnabotulinumtoxinA treatment. Nineteen patients (21%) were co-administered with a CM preventive drug (topiramate, amitriptyline, venlafaxine, paroxetine, propranolol, pizotifen, valproate acid, and pregabalin). The mean monthly medication intake was 33.2 (SD 5.6) and the use of NSAIDs and triptans was preponderant (only nine patients were on combination analgesics). See Table 1 for full baseline demographics and clinical characteristics. Side effects were reported by sixteen patients (19%) and consisted of neck pain (six patients), transient eyelid ptosis (four patients) and shoulders' tenderness (two patients). No serious adverse events were reported. Forty-four patients underwent three cycles of OnabotulinumtoxinA treatment, whereas thirty-four concluded a 12 months' treatment (five cycles). At the end of the third cycle patients were stratified into three groups according to reduction of headache days per month: >50% (responders), 30–50% (partial responders) and <30% (non-responders). See Figure 1 for patients' responder rates at 3, 6, 9, and 12 months of treatment.

**Table 1:** Subjects baseline demographic and clinical features.

	**Subjects (*n* = 84)**
Age, years (mean, SD)	48 (9.7)
Female, number (%)	61 (72.6%)
Disease duration, years (mean, SD)	10.1 (6.6)
Previous prophylaxis, number (mean, SD)	4.6 (2.6)
Add-on prophylaxis, number (%)	19 (21%)
Depressive symptoms, number (%)	36 (45%)
Anxiety symptoms, number (%)	44 (60%)
Medication overuse, number (%)	55 (65.5%)
NSAIDs, number (%)	11 (13%)
Triptans, number (%)	27 (30%)
Mixed analgesics, number (%)	17 (20%)
Analgesic consumption/month, mean (SD)	33.2 (5.6)
NSAIDs, mean (SD)	12.7 (18.8)
Triptans, mean (SD)	12.8 (15.8)
Headache days/month, mean (SD)	22.5 (5.9)
High intensity, mean (SD)	15.2 (8.9)
Low intensity, mean (SD)	7.2 (8.1)

When comparing baseline characteristics between responders and partial- and non-responders we found a significantly lower frequency of depressive symptoms and medication overuse in the responders' group (respectively *p* = 0.002 and *p* = 0.05; see Tables 2, 3).

**Table 2:** Frequency of depressive symptoms in responders, partial responders and non-responders.

		**Non-responders**	**Partial responders**	**Responders**
Absence depressive symptoms	Real	6	0	20
	Predicted	8.9	2.4	14.8
Presence depressive symptoms	Real	9	4	5
	Predicted	6.1	1.6	10.2

**Table 3:** Frequency of medication overuse (MO) in responders, partial responders and non-responders.

		**Non-responders**	**Partial responders**	**Responders**
Absence MO	Real	4	0	17
	Predicted	7.2	1.9	11.9
NSAIDs MO	Real	3	0	2
	Predicted	1.7	0.5	2.8
Triptans MO	Real	6	3	5
	Predicted	4.8	1.3	8
Mixed MO	Real	1	1	1
	Predicted	1	0.3	1.7

When comparing clinical and demographic characteristics between patients with or without depressive symptoms there was a significant difference in terms of analgesic consumptions, in agreement with the findings above reported (see Table 4). When conducting the same analysis comparing patients with or without medication overuse, lower MIDAS scores (104.9 ± 66.9 vs. 67.3 ± 51.4, *p* = 0.01), number of headache days (23.6 ± 5.9 vs. 20.4 ± 5.3, *p* = 0.01) and high intensity headache days per month (16.8 ± 9.4 vs. 12.2 ± 7.7, *p* = 0.01) at baseline were found in patients who did not present with medication overuse (see Table 5). Accordingly, with the diagnosis, patients without medication overuse also exhibited lower triptans (6.9 ± 6.07 vs. 15.9 ± 18.3, *p* = 0.01) and overall analgesics (17.6 ± 10.8 vs. 41.1 ± 37.04, *p* = 0.002) consumption compared to patients with medication overuse.

**Table 4:** Clinical and demographic characteristics in subjects with and without depressive symptoms.

	**Presence depressive symptoms**	**Absence depressive symptoms**	***p***
Age, years (mean, SD)	49.5 (8.5)	47.7 (10.6)	ns^#^
Female, number (%)	27 (75%)	32 (72.7%)	ns^§^
Disease duration, years (mean, SD)	10.6 (6.4)	9.8 (6.7)	ns^#^
Previous prophylaxis, number (mean, SD)	5.08 (2.9)	4.3 (2.4)	ns^#^
Add-on prophylaxis, number (%)	26 (72.2%)	14 (31.8%)	ns^§^
Medication overuse, number (%)	26 (70%)	25 (58%)	*p* < 0.05^§^
NSAIDs, number (%)	6 (16%)	4 (9.1%)	*p* < 0.05^§^
Triptans, number (%)	9 (25%)	17 (38.6%)	*p* < 0.05^§^
Mixed analgesics, number (%)	11 (30.5%)	4 (9.1%)	*p* < 0.05^§^
Analgesic consumption/month, mean (SD)	38.5 (39.4)	28.8 (27.1)	ns^#^
NSAIDs, mean (SD)	14.9 (19.9)	10.8 (18.3)	ns^#^
Triptans, mean (SD)	14.3 (21.4)	11.3 (9.8)	ns^#^
Headache days/month, mean (SD)	22.7 (6.4)	22.1 (5.8)	ns^#^
High intensity, mean (SD)	15.5 (9.3)	14.9 (8.5)	ns^#^
Low intensity, mean (SD)	7.2 (8.5)	7.2 (7.8)	ns^#^

# Mann-Whitney U test;

§ *Chi-squared test*.

**Table 5:** Clinical and demographic characteristics in subjects with and without medication overuse (MO).

	**Presence MO**	**Absence MO**	***p***
Age, years (mean, SD)	48.6 (9)	48 (11)	ns^#^
Female, number (%)	42 (76.4%)	20 (69%)	ns^§^
Disease duration, years (mean, SD)	9.7 (6.9)	10.8 (5.9)	ns^#^
Previous prophylaxis, number (mean, SD)	5.02 (2.8)	3.9 (1.8)	ns^§^
Depressive symptoms, number (%)	26 (51%)	10 (34.5%)	ns^§^
Anxiety symptoms, number (%)	29 (52%)	15 (51.7%)	ns^§^
Analgesic consumption/month, mean (SD)	41.1 (37)	14.6 (8.8)	*p* < 0.05^#^
NSAIDs, mean (SD)	14 (21.1)	10 (11.2)	ns^#^
Triptans, mean (SD)	15.9 (18.3)	3.93 (3.07)	*p* < 0.05^#^
Headache days/month, mean (SD)	23.6 (5.9)	20.4 (5.3)	*p* < 0.05^#^
High intensity, mean (SD)	16.8 (9.4)	12.2 (7.02)	*p* < 0.05^#^
Low intensity, mean (SD)	6.7 (9.1)	8.1 (5.8)	ns^#^

# Mann-Whitney U test;

§ *Chi-squared test*.

**Figure 2:**
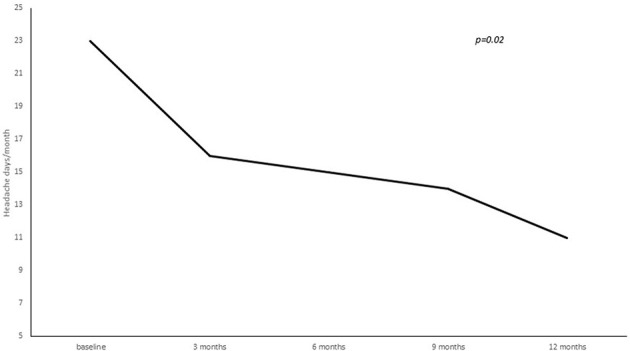
Number of headache days per month from baseline to 3, 6, 9, and 12 months of treatment.

**Figure 3:**
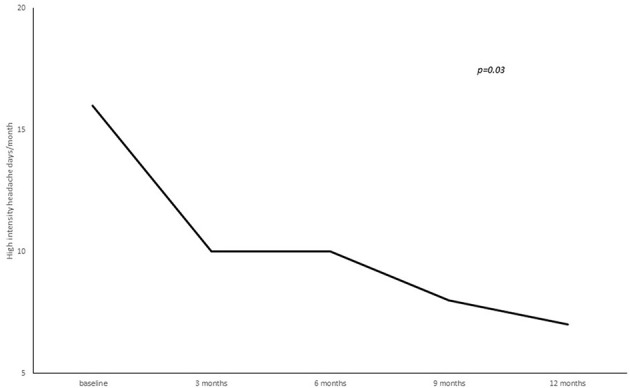
Number of high intensity headache days per month from baseline to 3, 6, 9, and 12 months of treatment.

**Figure 4:**
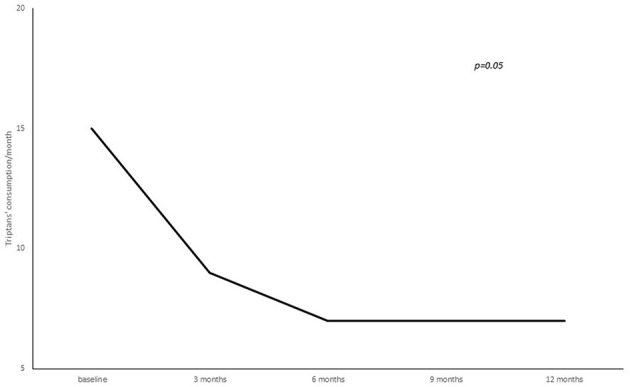
Number of triptans consumed per month from baseline to 3, 6, 9, and 12 months of treatment.

A statistically significant reduction from baseline to 3, 6, 9, and 12 months' treatment cycles in terms of total headaches days (22.8 ± 5.8 at baseline vs. 16.3 ± 7.8 at 3 months vs. 15.9 ± 8.1 at 6 months vs. 14.2 ± 7.9 at 9 months vs. 12.4 ± 7.06 at 12 months; *p* = 0.02), high intensity headaches days (15.4 ± 8.9 at baseline vs. 10.7 ± 8.9 at 3 months vs. 10.6 ± 7.5 at 6 months vs. 7.7 ± 7.9 at 9 months vs. 7.2 ± 6.1 at 12 months; *p* = 0.03) and triptans consumption (13.4 ± 16.4 at baseline vs. 9.2 ± 8.7 at 3 months vs. 7.2 ± 7.7 at 6 months vs. 8.2 ± 7.4 at 9 months vs. 7.7 ± 7.0 at 12 months; *p* = 0.05) per month was found (see Figures 2–4).

## Discussion

Our study confirms OnabotulinumtoxinA safety and efficacy in CM prophylaxis. Following three treatment cycles 47 and 20% of patients were classified as, respectively, responders and partial responders. A significant improvement in frequency, severity, and triptans consumption occurred from the first treatment cycle and was maintained throughout the study. These findings, in particular response percentages and the selective reduction of high intensity headache days and triptans consumption, is in line with results coming from previous larger studies (10–12).

Although numerous studies proved OnabotulinumtoxinA to be equally effective in the presence of medication overuse (18, 19) and depressive comorbidities (34, 35), they were found to be negative predictors in terms of treatment response. In fact, both medication overuse and depressive symptoms were less frequent in responders compared to partial and non-responders. Stating this does not imply that OnabotulinumtoxinA was not effective in treating CM in these subgroups of patients—both medication overuse and depressive symptoms were highly represented in our cohort—but they were, indeed, associated with a poorer outcome. In the present study, the subgroup who did not present medication overuse displayed significant baseline lower MIDAS scores, number of headache days, and high intensity headache days, compared to those who did present medication overuse. On the other side, when comparing patients with or without depressive comorbidity, a lower analgesic consumption was found in the latter.

Given the high comorbidity between CM and affective disorders and their association with negative treatment outcomes, we tested whether the presence of anxiety and depressive symptoms could predict OnabotulinumtoxinA efficacy. As a matter of fact, we did find a lower frequency of depression symptoms in the responders subgroup. Moreover, patients who did not present depressive symptoms not only had a higher probability to respond to OnabotulinumtoxinA, but also presented, at baseline, with a lower analgesic intake in the absence of a lower headache frequency compared to the other group.

Mood and anxiety disorders are two to ten times more frequent in migraineurs compared to the general population, especially in chronic migraineurs (5). Based on current evidence, the relationship between migraine and psychiatric comorbidities seems bidirectional, with each condition increasing the incidence of the other (36). The recognition of these comorbidities is essential for several reasons, i.e., diagnostic vigilance, treatment options and implications regarding outcomes and adherences. Several pathophysiological mechanisms have been proposed to explain this comorbidity. These include genetic factors, monoamines dysfunctions, ovarian hormone and hypothalamic-pituitary adrenal axis dysregulation (36). Serotoninergic deficits are implicated in the pathogenesis of both migraines and depression. Reduced serotonin levels have been documented during migraine attacks and serotonin depletion has been found to enhance cortical spreading depression-induced trigeminal nociception by increasing the cortical excitability and sensitivity of trigeminal nociceptive system (37). Although less consistently, genetic studies also support a plausible role of the dopaminergic system in migraine and depression, with different polymorphisms in the dopamine transporter and D2–D4 receptors being more frequent in subjects suffering from both migraines and depression compared to the general population (38). Dopaminergic dysfunctions might be involved in the activation and sensitization of the trigeminovascular system (38). Twin and family studies demonstrated that around 20% of the variance in migraine and depression is due to shared genetics (39). Moreover, it has been found that migraineurs with comorbid depression display smaller total brain volumes compared to patients suffering from either migraine alone or depression alone (40). Taken all together, these findings suggest a significant shared background between migraine and depression and it has been proposed that migraine with and migraine without depression comorbidity might represent two distinct clinical and biological phenotypes (41, 42). Given our results, the “pure migraine” phenotype demonstrated a better response pattern to OnabotulinumtoxinA. Why would that be the case? Depression, along with other factors, is characterized by a series of neurotransmitter events and brain areas repeated activation that leads to a state of central sensitization. Chronic migraine is equally characterized by central sensitization, with migraine chronification being significantly influenced by medication overuse and depression. Thus, it seems plausible that patients presenting all these conditions might exhibit a well-consolidated and more severe state of central sensitization, making treatment challenging, independent from disease duration or headache frequency.

To our knowledge, three previous studies have assessed OnabotulinumtoxinA in CM with comorbid depression (32, 34, 43). Boudreau et al. (34) found a significant improvement in both number of headache days and depressive symptoms following 24 weeks of treatment, and a recent study by Blumenfeld et al. (35) found a significant improvement in depressive symptoms in the overall cohort and an even better outcome in responders compared to non-responders. However, these studies were not designed to analyze response predictors', thus, we do not know whether responders exhibited a higher or lower level of depressive symptomatology compared to non-responders. Disco et al. (32), in line with our results, also found depression and anxiety disorders to be associated to a lower responsiveness trend at the limit of significance.

Treatment-wise our results might suggest that, in the everyday setting, patients displaying significant depressive traits might find a more beneficial outcome from other oral therapeutics (e.g., amitriptyline or topiramate) alone or in association with OnabotulinumtoxinA and, in the presence of medication overuse, withdrawal treatments preceding treatment might have long-term benefits. A more stringent selection of patients presenting a “pure migraine” phenotype could improve the identification of OnabotulinumtoxinA responders, allowing a more tailored treatment for CM, making it a double win for both patients and clinicians.

This study has several potential limitations. Firstly, the small sample size. Although our efficacy results are in agreement with larger, multi-center studies like the PREEMPT and COMPEL studies, conclusions regarding response predictors' need further replications. Secondly, depressive and anxiety symptoms were assessed qualitatively, i.e., no validated scales were used, although collected in the form of open questions regarding the diagnostic items formulated by the DSM-V (43).

## Data Availability

The raw data supporting the conclusions of this manuscript will be made available by the authors, without undue reservation, to any qualified researcher.

## Ethics Statement

The research protocol was approved by the Ethics Committee of the Brescia Hospital, Brescia, Italy. Written informed consent was obtained from all participants.

## Author Contributions

FS: study conception and design, acquisition of data, analysis and interpretation of data, and drafting of manuscript. SC: acquisition of data, analysis and interpretation of data, and drafting of manuscript. PL: acquisition of data and critical revision. RR: study conception and design, acquisition of data, drafting of manuscript, and critical revision. AP: drafting of manuscript and critical revision.

### Conflict of Interest Statement

The authors declare that the research was conducted in the absence of any commercial or financial relationships that could be construed as a potential conflict of interest.

## Abbreviations

CM: chronic migraine
EM: episodic migraine
MIDAS: Migraine Disability Assessment Score
NSAIDs: non-steroidal anti-inflammatory drugs
SF-36: Short Form-36.
